# The Incidence of Type-1 Diabetes in NOD Mice Is Modulated by Restricted Flora Not Germ-Free Conditions

**DOI:** 10.1371/journal.pone.0017049

**Published:** 2011-02-25

**Authors:** Cecile King, Nora Sarvetnick

**Affiliations:** Department of Immunology, The Scripps Research Institute, La Jolla, California, United States of America; La Jolla Institute of Allergy and Immunology, United States of America

## Abstract

In the NOD mouse, the incidence of type-1 diabetes is thought to be influenced by the degree of cleanliness of the mouse colony. Studies collectively demonstrate that exposure to bacterial antigen or infection in the neonatal period prevents diabetes [1], [2], [3], [4], [5], [6], [7], [8], [9], [10], supporting the notion that immunostimulation can benefit the maturation of the postnatal immune system [11]. A widely accepted extrapolation from this data has been the notion that NOD mice maintained under germ-free conditions have an increased incidence of diabetes. However, evidence supporting this influential concept is surprisingly limited [12]. In this study, we demonstrate that the incidence of diabetes in female NOD mice remained unchanged under germ-free conditions. By contrast, a spontaneous monoculture with a gram-positive aerobic spore-forming rod delayed the onset and reduced the incidence of diabetes. These findings challenge the view that germ-free NOD mice have increased diabetes incidence and demonstrate that modulation of intestinal microbiota can prevent the development of type-1 diabetes.

## Introduction

The development of the postnatal immune system is guided by the interactions of lymphocytes with self-MHC/peptide ligands derived from our body's own tissues and those from the environment, such as the commensal microbial flora of the gastrointestinal tract and the diet. The important role of the gastrointestinal microbiota has been emphasized by the evidence of reduced intestinal lymphatic tissue and underdeveloped lymphoid organs in germ-free mice [13], [14], [15], [16]. The interplay between the host immune system and commensal bacteria is dynamic and continuous since the size and cellularity of gastrointestinal lymphoid tissues recover following selective colonization of germ-free animals [13], [14]. Studies on local immune function demonstrate that IgA secreting plasmablasts are reduced in germ free mice [17] and the induction of commensal specific IgA [18] has been shown to occur in response to current bacterial exposure [19]. The molecular basis for the influence of commensal bacteria on host immune function remains incompletely understood, but recent studies have revealed a critical role for the nucleotide-binding oligomerization domain containing 1 (NOD1) protein [20] and bacterial polysaccharide [21].

The way in which antigenic stimulation guides the development and maintenance of a healthy immune system is of fundamental importance to our understanding of immunological tolerance. In the non-obese diabetic (NOD) mouse strain, the target pancreatic insulin producing beta cells are attacked and destroyed by activated immune cells, leading to type-1 diabetes. Several infectious, and non-infectious agents are known to prevent type-1 diabetes in NOD mice. They include; persistent viral infection (MHV [1], LCMV [2]), mycobacterial infection [3], bacterial antigens [4], [5]; Hsp65 [6], [7], [8] and complete (heat-killed mycobacterium-containing) Freunds adjuvant (CFA) [9], [10]. Stimulation with adjuvant containing bacterial extracts in the neonatal period is known to prevent diabetes and imparts qualitative and quantitative changes in the immune cell compartments that lasts throughout adulthood [9], [22]. There has been a number of hypotheses presented that could account for the protective effect of immunostimulation such as a change in the cytokine milieu [23] and the increase in T cell numbers or populations of regulatory T cells [5], [22], [24], [25], [26].

Thus, the incidence of type-1 diabetes in NOD mice is thought to reflect the degree of cleanliness of the colony. A widely accepted extrapolation from these data has been that NOD mice maintained under germ-free conditions have an increased incidence of diabetes. However, there is little evidence to support this view [12].

## Materials and Methods

A germfree caesarean derivation was performed on NOD mice using 10 females and 5 males. Ceasarean-derived mice were weaned onto sterile dams and housed in sterile isolators to maintain germfree status and fed an endotoxin free NIH31-M diet, designed to provide appropriate nutrition after being autoclaved http://www.taconic.com/wmspage.cfm?parm1=292 until thirty weeks of age at Taconic Farms breeding facility, Germantown, NY, USA. All supplies were sterilized and entered into the isolator using strict aseptic techniques. Mice underwent monthly microbiological testing to ensure that germfree rodents were free of aerobic and anaerobic organisms (for a complete list of agents see http://www.taconic.com/wmspage.cfm?parm1=265). Urine glucose was measured every one or two weeks and mice with frank glucosuria -two consecutive reading of 4+ (estimated at 55 mmol/L urine glucose) were considered diabetic. Control female NOD mice were housed under specific pathogen-free (SPF) conditions at Taconic and the Scripps rodent colony, which were handled in accordance with the TSRI Animal Care and Use Committee, which approved this study (A3194-01). Blood glucose values (BGV) were determined using Glucofilm blood glucose strips (Miles Diagnostic, Elkhart, IN). Mice were considered diabetic following two consecutive blood glucose readings above 18 mmol/L.

## Results and Discussion

**Figure 1:**
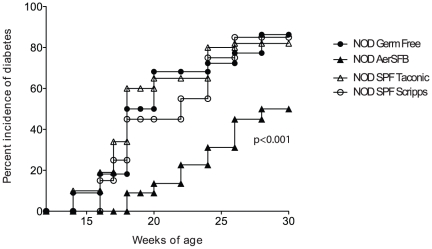
The incidence of type-1 diabetes in female NOD mice remains unchanged under germ-free conditions but is reduced by restricted flora. Cumulative diabetes incidence of germ-free female NOD mice (NOD germ-free n = 22), female NOD mice monocolonized with an aerobic spore-forming bacteria (*Bacillus cereus*) detected at week 16 (NOD AerSFB n = 22) and female NOD mice housed under specific pathogen-free conditions (NOD SPF Scripps n = 22, NOD SPF Taconic n = 40). Urine glucose was measured every one or two weeks as shown and mice that had reached 4+ (55 mmol/L) were considered diabetic. There was a significant inhibition (p<0.001) in the incidence of diabetes in monocolonized NOD mice compared with SPF NOD mice, but no significant difference between germ free and SPF NOD mice (p>0.05), Anova.

We sought to determine the influence of intestinal microbiota on the incidence of type-1 diabetes in NOD mice. To our surprise, the incidence of type-1 diabetes in female germ free NOD mice (n = 22) was indistinguishable from that of NOD mice housed under SPF conditions in our colony (n = 20) (Figure 1, p>0.6696) or the 80% incidence of female NOD mice housed under SPF conditions at Taconic (Figure 1, p<0.78). However, our findings also indicated that intestinal microflora had the capacity to influence the development of type-1 diabetes. In one cohort of NOD mice (n = 22) housed in a separate isolator, a spontaneous contamination with a gram-positive aerobic spore-forming rod (that was subsequently typed as *Bacillus cereus*) was detected at week 16. These mice exhibited a delayed onset, and reduced incidence of clinical disease (p<0.001) during the 30-week study period (Figure 1).

The NOD genetic background is one important factor contributing to the normal development of T1D in female NOD mice in the absence of known intestinal microbiota. However, the critical influence of the environment was highlighted by the reduced incidence of diabetes following spontaneous monoculture with aerobic, spore-forming, bacteria. Diet has a well-established role in the development of T1D in NOD mice [27], [28], [29] and the observed reduction of diabetes is likely to have reflected diabetes promoting as well as regulatory factors from the interactions of dietary components, restricted microbiota and the immune system. The extent to which the monoculture influenced the development of immune regulatory networks, as has been suggested in *MyD88-/-* NOD mice [30], remains unknown. However, since restricted flora limit the growth of other organisms through local competition, our findings did not exclude the possibility that germ-free mice harbour microorganisms that play a role in diabetes development but defy current methods of detection.

Taken together, these findings debunk the myth that germ-free NOD female mice have increased diabetes incidence and support the notion that modulation of intestinal microbiota can have beneficial effects on the development of autoimmune diabetes. Future examination of the influence of an array of commensal microbiota on diabetes incidence offers the potential for non-invasive approaches for individuals at risk of developing type-1 diabetes.
